# Modeling Thermal Suitability for Reindeer (*Rangifer tarandus* ssp.) Brainworm (*Elaphostrongylus rangiferi)* Transmission in Fennoscandia

**DOI:** 10.3389/fvets.2020.603990

**Published:** 2021-01-15

**Authors:** Hannah Rose Vineer, Torill Mørk, Diana J. Williams, Rebecca K. Davidson

**Affiliations:** ^1^Department of Infection Biology and Microbiomes, Institute of Infection, Veterinary and Ecological Sciences, University of Liverpool, Liverpool, United Kingdom; ^2^Department of Animal Health and Food Safety, Norwegian Veterinary Institute, Tromsø, Norway

**Keywords:** elaphostrongylus, reindeer, rangifer, climate, model, parasite, wildlife-livestock, degree-day

## Abstract

The brainworm*, Elaphostrongylus rangiferi*, is a nematode which causes neurological disorders (elaphostrongylosis) in reindeer (*Rangifer tarandus* ssp.). Favorable climatic conditions have been inferred as the cause of sporadic outbreaks of elaphostrongylosis in Norway, supported by positive associations between observed outbreaks/intensity of infection and summer temperatures in the previous years. Climate warming which results in increased transmission of *E. rangiferi* therefore presents a risk to the health of semi-domesticated and wild reindeer in Fennoscandia (Norway, Sweden, and Finland), the health of co-grazing small ruminants, and the livelihoods of indigenous Sámi herders. As a first step toward developing climate change impact assessments for *E. rangiferi*, a degree-day model was developed for larval development in a range of gastropod hosts and applied to historic weather data. Predictions were validated by statistical and qualitative comparison against historic parasitological and outbreak records. The model predicted an overall increase in thermal suitability for *E. rangiferi*, which was statistically significant in the north and along the Scandinavian mountain ranges, where reindeer density is highest. In these regions annual cumulative temperature conditions are suitable for larval development within a single year, potentially changing *E. rangiferi* epidemiology from a 2-year transmission cycle to a 1-year transmission cycle. This is the first mechanistic model developed for *E. rangiferi* and could be used to inform veterinary risk assessments on a broad spatial scale. Limitations and further developments are discussed.

## Introduction

The brainworm*, Elaphostrongylus rangiferi*, is a nematode which causes neurological disorders (elaphostrongylosis) in reindeer (*Rangifer tarandus* ssp.). Similar to other protostrongylid nematodes, *E. rangiferi* relies on a gastropod intermediate host for development from the first larval stage (L1) shed by infected reindeer to the third, infective larval stage (L3). Development within the ectothermic gastropod host is highly temperature-dependent (1). As a result, the rate of development and transmission varies with ambient temperature.

Favorable climatic conditions have been inferred as the cause of sporadic outbreaks of elaphostrongylosis in Norway [reviewed by (2)]. This is supported by a comparison of summer temperatures in years with and without outbreaks [1960-1993; (3)], and a positive association between L1 abundance in feces and summer temperature in the previous year [1974-1988; (4)].

Peak L1 excretion by infected reindeer occurs during the spring (4). These L1 then infect gastropod intermediate hosts and the majority overwinter in their gastropod hosts, completing development to L3 the following year. When conditions allow, development to L3 can be accelerated, with development of larvae that have overwintered in the intermediate host completed earlier in spring, and even as early as August the same year (i.e., overwintering not required). This could lead to higher incidences of disease and diagnoses of elaphostrongylosis earlier in the year [reviewed by (2)]. Worryingly, average increases in temperature of 0.5–0.8 °C between 1959 and 2018 have been recorded within the ranges of 6 populations of wild reindeer in Norway, and prevalence and observed intensity of infection increased with increasing temperature and decreasing altitude (5). Such climate warming presents a risk to the health of semi-domesticated and wild reindeer, and of co-grazing small ruminants (6).

Increased transmission of *E. rangiferi* also presents a risk to the livelihoods of indigenous Sámi herders, who must also contend with land use disputes and low profit margins (7). Reindeer are an integral part of Fennoscandian cultural identity and ecosystems. Wild mountain reindeer (*R. t. tarandus*) are present throughout the higher altitude regions of southern Norway, and wild forest reindeer (*R. t. fenniscus*) are found in pockets of central and southern Finland (8). These wild reindeer exist alongside semi-domesticated reindeer, which are managed across up to 40% of the area of Fennoscandia (7). In southern Norway, a small number of non-Sámi herds are managed, whereas throughout mid-north Norway and Sweden, reindeer are herded by the indigenous Sámi people (a right protected by law), completing seasonal migrations between summer and winter grazing areas each year. By contrast, in mid-north Finland, long-distance seasonal migrations are no longer practiced and herding is not restricted to Sámi people only (8).

Little is known about the current distribution, prevalence and intensity of *E. rangiferi* infection in Swedish and Finnish reindeer (2), although there is a high level of awareness of *E. rangiferi* among Swedish herders (9). Similarly, there is a lack of data regarding current control methods employed by herders even though some macrocyclic lactones are licensed for use in reindeer in all three countries for the treatment of warbles (2). The lack of systematic records on the prevalence and frequency of anthelmintic use at herd level, hinders comparison of treatment differences within, and between, herds and regions.

In light of the importance of reindeer to Sámi culture and livelihoods, the potential transmission to farmed small ruminants, and recent ongoing elaphostrongylosis outbreaks in Norway (2, 10), there is a need to improve understanding of how climate change might impact on reindeer health and productivity through altered seasonal forage and disease dynamics to enable mitigation strategies to be put in place.

As a first step toward developing climate change impact assessments for *E. rangiferi*, a degree-day model was developed for L1-L3 development in a range of gastropod hosts and applied to historic weather data. The aims were to (1) evaluate whether climate (thermal) suitability for *E. rangiferi* development in its intermediate hosts has changed in Fennoscandia in recent decades and (2) provide current estimates of thermal suitability that can be used as a basis for veterinary risk assessment of this understudied parasite.

## Methods

All analyses were completed in R software (11). The following R packages were used for data visualization: *rnaturalearthdata* (12), *raster* (13), *gplots* (14), *sf* (15), *ggplot2* (16), and *cruts* (17). Readers are referred to the online version for color figures, but the *viridis* color schemes used to plot model output are designed to print true to scale in black and white.

### Temperature-Dependent Larval Development in the Intermediate Host

To parameterize the model, data detailing the time to development from L1-L3 in a range of gastropod hosts and at a range of temperatures was required. Skorping and Halvorsen (18) experimentally observed the development of L1-L3 in two gastropods, *Arianta arbustorum* and *Euconulus fulvus*, at a range of constant temperatures. Skorping (19) observed the development of L1-L3 in a range of gastropod species at 20°C. Skorping (20) observed the development of L1-L3 in *Arianta arbostorum* at 20°C. For each of these studies, and for each group within a study, the number of days post-infection when L3 were first observed was noted. Where a range of sampling days post-infection were reported, the smallest figure was used. Halvorsen and Skorping (1) also noted that development was not observed at 8°C, and that there was an increase in snail mortality at 24°C and 28°C.

### Degree-Day Model

A simple degree-day model was developed for *E. rangiferi* development from L1-L3, following the methods described by Kutz et al. (21) for the protostrongylid nematode *Umingmakstrongylus pallikuukensis*. Degree-day models estimate the number of heat units accumulated over time, under the assumption that there is a positive linear relationship between temperature and development, and a minimum threshold for development.

The number of days post-infection (d.p.i.) that L3 were first observed in a gastropod host was used to calculate the accumulated degree-days (DD) using the temperature of the observation (T), the minimum temperature threshold for development [T_min_; 8°C; (1)] and the formula:

(1) $$\begin{array}{r} DD\;=\;T-T_{\min}\;\times \;d.p.i. \end{array}$$

This basic model was used to estimate the degree-days required to complete development from L1-L3 in the gastropod hosts housed in controlled conditions (see section Temperature-Dependent Larval Development in the Intermediate Host). However, free-living gastropods typically have the freedom to select optimal environmental conditions. To account for behavioral thermoregulation by the gastropod host, Kutz et al. (21) replaced all values for T >21°C with 21°C. Halvorsen and Skorping (1) noted an increase in mortality of *A. arbustorum* and *E. fulvus* at 24°C and 28°C. Mortality rates were unaffected at 20°C. We therefore assumed that snails would seek cooler temperatures when ambient temperatures increased above ~21°C and adopted the same threshold approach as Kutz et al. (21) for all projections using the degree-day model.

### Model Projections

Mean daily air temperature data for the period 1950-2019 were obtained from the EOBS gridded dataset [version 21.0e; (22)] as a NetCDF (.nc) file which was read into R as a three-dimensional array using the *ncdf4* package (23). Shape files delineating country boundaries were obtained from Eurostat (ec.europa.eu). Fill values were replaced with “NA” and daily temperatures above 21°C were replaced with 21°C to account for the intermediate host's potential behavioral thermoregulation. Equation 1 was then applied to each cell and day in the gridded dataset to estimate the number of degree-days accumulated each day. Data were disaggregated by year, and the total accumulated degree-days calculated for each grid cell and each year between 1950 and 2019.

Annual degree-day estimates were then divided by the degree-days required to complete development from L1-L3 (see section Temperature-Dependent Larval Development in the Intermediate Host and Degree-Day Model) to obtain an index of thermal suitability. In arthropod species where the entire life cycle is completed under ambient conditions, the Thermal Suitability Index would broadly represent the number of successive generations that can be completed per year. However, due to the indirect life cycle of *E. rangiferi* and the extended prepatent period in the ungulate definitive host (5), the Thermal Suitability Index is used as an indication of the number of years required for transmission. Thermal Suitability Indices >1 indicate that L1-L3 development can be completed within 1 year and Thermal Suitability Indices <1 indicate that it would take more than 1 year to complete development from L1-L3. Thus, a change from an index of <1 to an index of >1 over time represents a change in parasite epidemiology, from a 2-year transmission cycle to a 1-year transmission cycle.

A mean annual thermal suitability index was calculated for the entire region within the boundaries of Norway, Sweden, and Finland, and a Spearman rank correlation applied to the data to assess general trends in climatic suitability over time (since time is ordinal, requiring a non-parametric test).

Kendall Tau was used to assess spatial trends in Thermal Suitability Indices over time, using the *spatialEco* R package (24), which returns pixel-by-pixel slopes of the association between Thermal Suitability Indices and year, a correlation statistic (tau) and *p*-values. The slope indicates the direction of the trend (positive values indicate an increase in thermal suitability over time). Tau indicates the strength of the correlation between thermal suitability and time, and the *p*-value indicates statistical significance, with *p* < 0.05 indicating statistically significant trends.

The mean Thermal Suitability Index for the entire region, and pixel-by-pixel were calculated and deducted from annual mean (entire region) and pixel Thermal Suitability Indices, respectively, to obtain Thermal Suitability Index anomalies. These anomalies enable positive and negative trends relative to the overall mean to be visualized.

### Model Validation

The model was validated using L1 abundance data collected in spring each year from two herds in northern Norway (Porsanger fjord) between 1974 and 1988 (4). Mean L1 abundance for calves (<1 years old), adults (>2 years old), and young (1-2 years old) reindeer, were extracted to the nearest 25 larvae per gram from the relevant figures in the supplementary material accompanying Halvorsen (4). Approximate coordinates for the two herds were also obtained based on the accompanying maps. Although adult and young reindeer may have been infected during the spring and summer of the previous 2 years, and the calves only infected during the previous year, in their original cross correlation time series analyses, Halvorsen found no evidence of a significant time lag. Therefore, Thermal Suitability Indices for the previous year only were extracted to match each L1 abundance observation. A hierarchical linear mixed model was implemented using the *lmerTest* package (25) and used to evaluate the relationship between the log-transformed L1 abundance and scaled Thermal Suitability Index in the previous year. Age class of the animal and herd were input as nested random effects (age within herd). Predictions were extracted from the fitted model for visualization using the *ggeffects* package (26).

Other georeferenced, quantitative data were not available for model validation. Therefore, spatiotemporal patterns were also compared against qualitative descriptions of outbreaks and regional patterns of infection, as reviewed by Davidson et al. (2) and described by Handeland et al. (5).

## Results

### Temperature-Dependent Larval Development in the Intermediate Host

A total of 24 point estimates of time required for development at temperatures between 12 and 28°C were extracted from the literature (Table 1). The data included a range of gastropod host species and a range of infection intensities, and as a result the number of d.p.i. to first observation of L3 varied widely between 12 and 75 days (median = 19 days). There was potentially significant measurement error as a result of the sampling interval, which ranged between 3 and 20 days.

**Table 1:** Temperature-dependent development data extracted from the literature, and estimated degree-days required for development from L1-L3 based on these data.

**Gastropod species**	**Temperature (°C)**	**L3 first observed** ** (days post-infection)**	**Sampling interval** ** (temporal measurement error)**	**Source**	**Degree-days**
*Euconulus fulvus*	12	60	20	(1)	240
*Euconulus fulvus*	16	30	14	(1)	240
*Euconulus fulvus*	20	20	5	(1)	240
*Euconulus fulvus*	24	15	3	(1)	240
*Euconulus fulvus*	28	15	3	(1)	300
*Arianta arbustorum*	12	75	15	(1)	300
*Arianta arbustorum*	16	35	5	(1)	280
*Arianta arbustorum*	20	20	5	(1)	240
*Arianta arbustorum*	24	15	3	(1)	240
*Arianta arbustorum*	28	12	3	(1)	240
*Succinea pfeifferi*	20	18	6	(19)	216
*Cochlicopa lubrica*	20	24	6	(19)	288
*Discus ruderatus*	20	18	6	(19)	216
*Arion subfuscus*	20	18	6	(19)	216
*Arion hortensis*	20	18	6	(19)	216
*Deroceras reticulatum*	20	12	6	(19)	144
*Deroceras laeve*	20	18	6	(19)	216
*Euconulus fulvus*	20	18	6	(19)	216
*Clausilia bidentata*	20	24	6	(19)	288
*Arianta arbustorum*	20	18	6	(19)	216
*Arianta arbustorum*	20	21	7	(20)	252
*Arianta arbustorum*	20	21	7	(20)	252
*Arianta arbustorum*	20	21	7	(20)	252
*Arianta arbustorum*	20	28	7	(20)	336
	**Median (range)**	**19 (12–75)**		**Mean (standard deviation)**	**245.17 (39.38)**

### Degree-Days and Model Projections

*E. rangifer* required an average of 245 degree-days (S.D. 39) to complete development from L1-L3 (Table 1).

**Figure 1:**
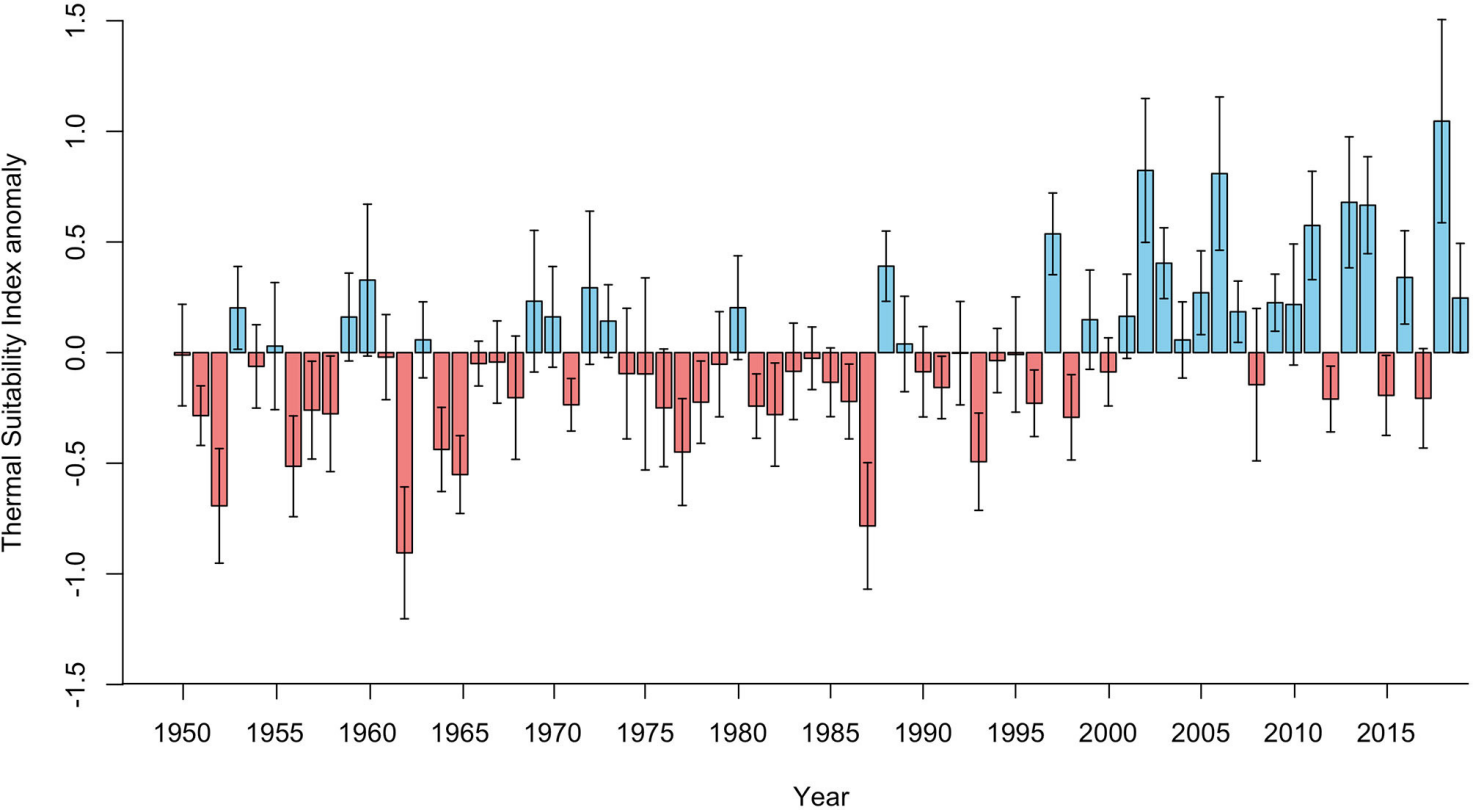
Annual Thermal Suitability Index anomalies for the entire Fennoscandian region (bars = mean, whiskers = standard deviation), estimated by deducting the annual mean Thermal Suitability Index from the corresponding mean for the entire period (1950-2019). Red bars indicate lower than average Thermal Suitability Indices, and blue bars indicate higher than average Thermal Suitability Indices.

**Figure 2:**
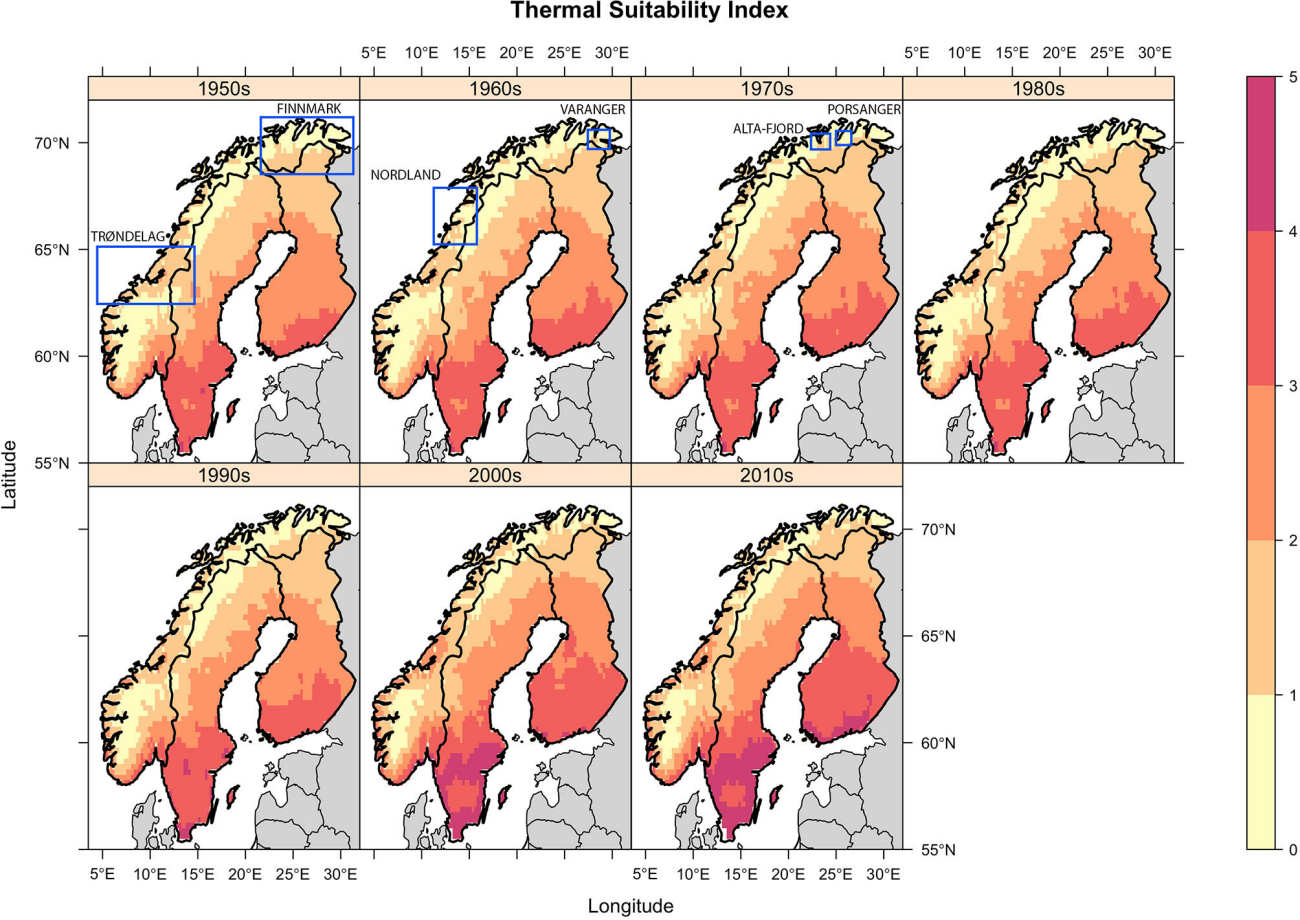
Decadal mean Thermal Suitability Indices for *E. rangiferi* development from L1-L3 in Fennoscandia. Indices >1 indicate that development can take place within a year. Indices <1 indicates that developing larvae would overwinter in infected gastropods or as L1 larvae in the environment and continue development the following spring. Regions referred to for model validation are labeled and demarcated with a blue rectangle. Note that in order to ensure the figure is legible it was not possible to place the rectangles in the panels corresponding to the period in which the observations were made. Therefore, the rectangles do not convey temporal information.

There was a significant positive correlation between average annual Thermal Suitability Indices and time (S = 33712, rho = 0.41, *p* < 0.001). Fifteen of the previous 20 years (2000–2019) yielded higher than average Thermal Suitability Indices, compared with just 6 out of 20 between 1950 and 1969 (positive anomaly values; Figure 1). However, there was substantial interdecadal (Figure 2) and interannual variability (Figure 1).

**Figure 3:**
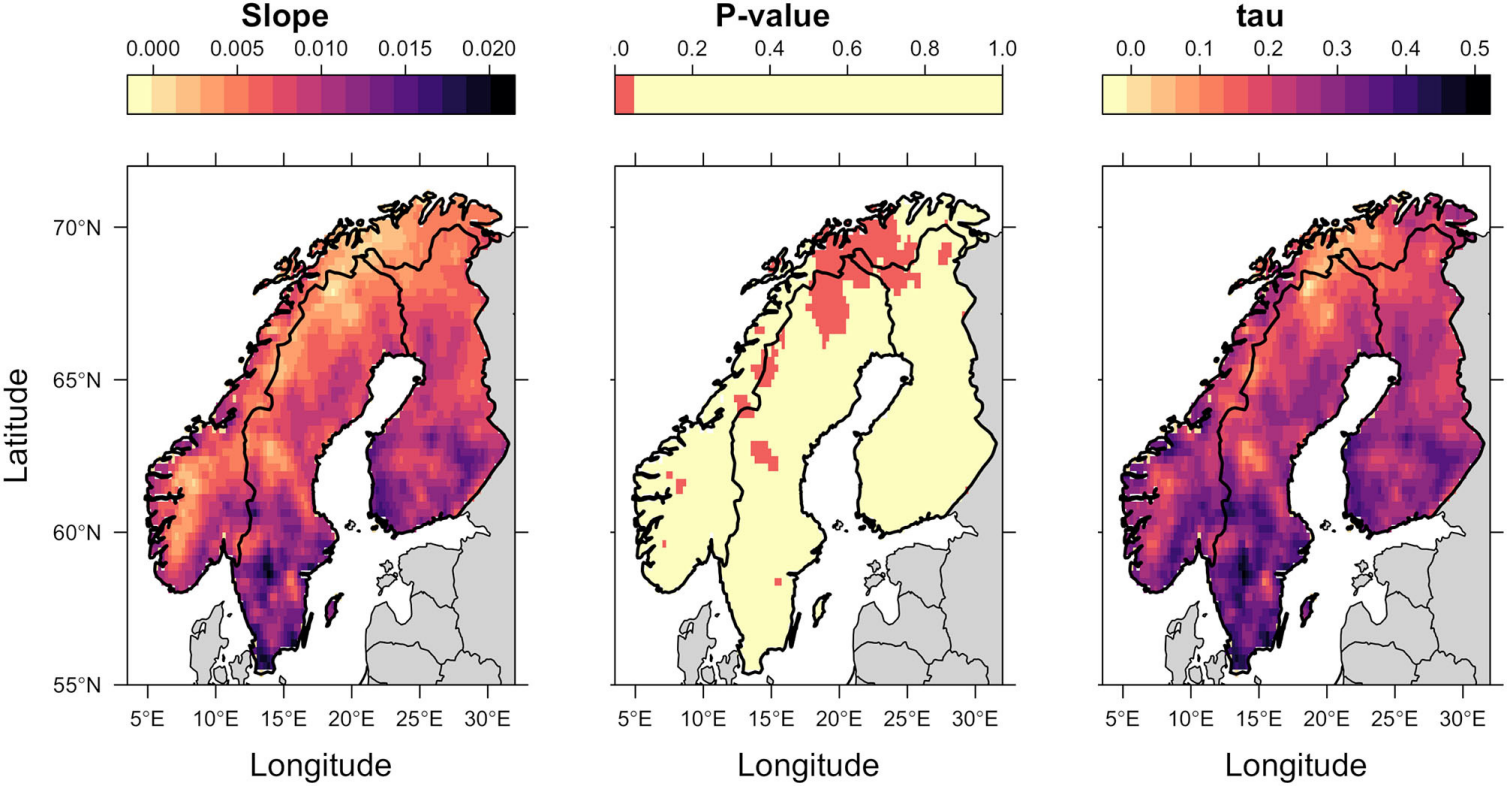
Kendall Tau analysis of spatial trends in annual average Thermal Suitability Indices over time. The slope indicates the direction of the trend (positive values indicate an increase in thermal suitability over time). Tau indicates the strength of the correlation between thermal suitability and time, and the *p*-value, shown using a binary color scheme, indicates statistical significance, with *p* < 0.05 (red) indicating statistically significant trends.

Spatial trends were heterogeneous. There was a trend of increasing thermal suitability (Figure 3; slope panel) and a positive correlation between thermal suitability and time (Figure 3; tau panel). There were statistically significant increases in predicted Thermal Suitability Indices along the Scandinavian mountain range bordering Norway and Sweden, and throughout Finnmark county (northern Norway; Figure 3). This can be seen as a contraction in the area where Thermal Suitability Indices were <1 (Figure 2).

Although the greatest predicted increases in thermal suitability for *E. rangiferi* were located in southern regions, outside of the ranges of wild and semi-domesticated reindeer (Figures 2, 3, slope and tau (correlation) panels), this was not statistically significant (Figure 3, *p*-value panel).

The majority of Sweden and Finland had high predicted levels of climatic suitability, with only the northernmost (higher elevation) regions recording Thermal Suitability Indices <1. The southern Norwegian regions occupied by mountain reindeer and non-Sámi herds largely remained unsuitable for development from L1-L3 within a single year (Figure 2). Although, there was a contraction of the area where Thermal Suitability Indices were <1, which could affect some populations of wild and herded reindeer (e.g., between Trøndelag, Røros and Hedmark, along the southern border with Sweden; Figure 2), this was for the most part not statistically significant (Figure 3, *p*-value panel).

### Model Validation

**Figure 4:**
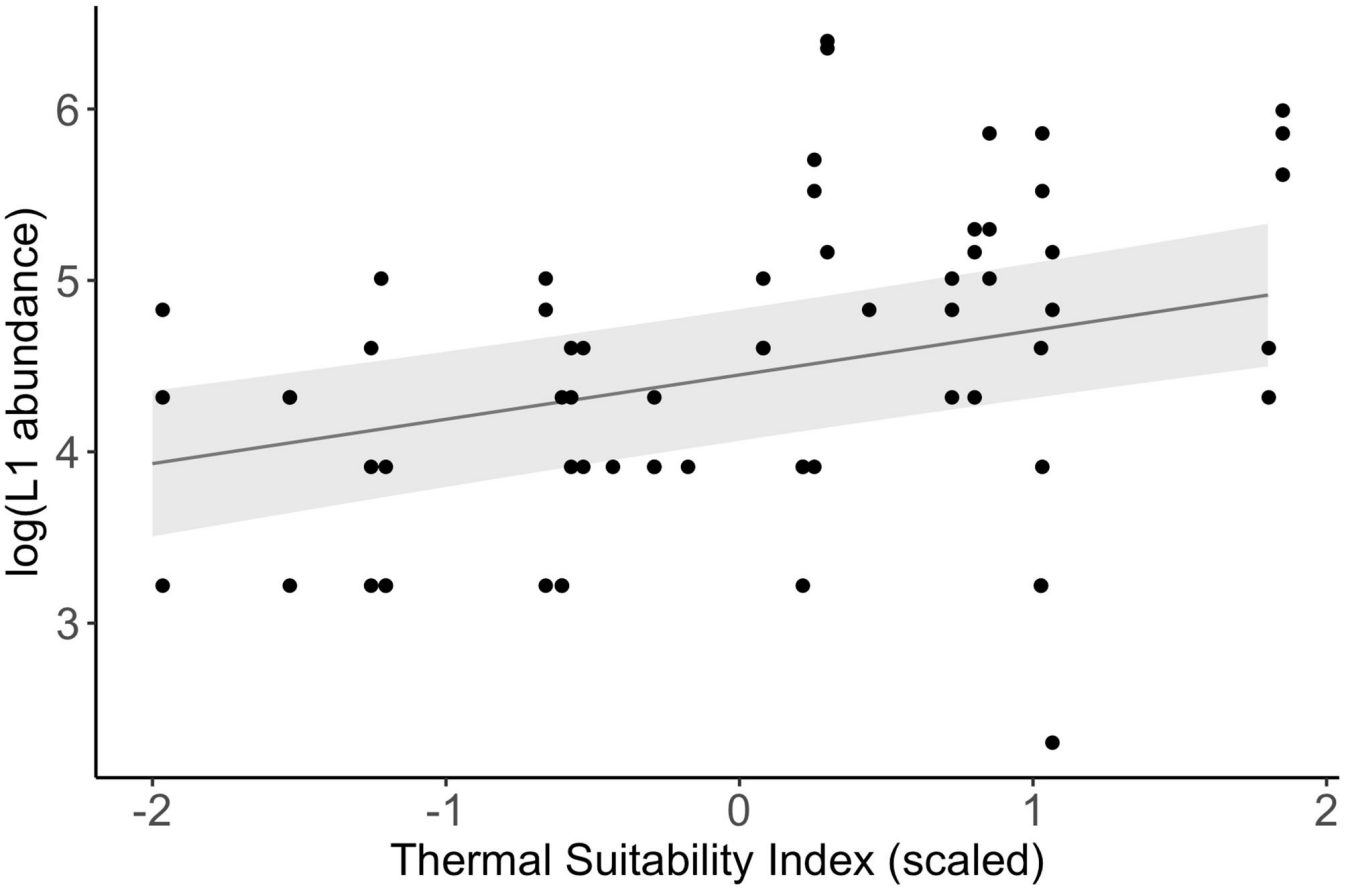
Relationship between log-transformed L1 abundance measured in spring between 1974 and 1988 (4) and Thermal Suitability Indices in the previous year (points). The line and shaded area represent a linear mixed model fitted to the data, and the standard error, respectively.

There was a significant positive association between log-transformed L1 abundance and Thermal Suitability Indices in the previous year at Porsanger fjord, northern Norway (fixed effect estimate = 0.26 (S.E. = 0.09), F_1, 58_ = 8.18, *p* = 0.006; Figure 4).

Qualitative comparisons against historic reports of outbreaks reviewed by Davidson et al. (2) also tended to support the model predictions. The paucity of historic records of elaphostrongylosis in Sweden and Finland was at odds with the high thermal suitability predicted throughout these countries. However, within Norway, historic observations supported model predictions. Specifically, outbreaks in the south Varanger region in the early 1960s, in Finnmark in the 1970s, and in Trøndelag in the past decade were predicted by elevated thermal suitability during these periods (Figure 2). Spatial heterogeneity in observed *E. rangiferi* abundance in the Alta-Fjord area and surrounding region in the 1960s are partially reflected in model predictions (Figure 2; 1960s panel). Further details of these historic outbreaks are discussed in section Discussion.

## Discussion

A degree-day model was developed to predict spatio-temporal thermal suitability for *E. rangiferi* transmission, based on interpolated temperature datasets and laboratory observations of temperature-dependent *E. rangiferi* larval development in its gastropod intermediate hosts. The model predicted a heterogeneous distribution of thermal suitability throughout Fennoscandia, which was supported by historic observations of intensity of infection and elaphostrongylosis in Norway. Therefore, an increase in thermal suitability predicts an increase in intensity of infection the following year. There was a trend toward increasing thermal suitability over time, which was statistically significant in northern Norway and along the Scandinavian mountain range bordering Norway and Sweden. Based on the model and temperature data for 2010-2019, there is currently an elevated transmission risk along the Norwegian coast, the Norway-Finland border, and throughout Sweden and Norway. In some areas of northern Norway and the edges of the Scandinavian mountain ranges there has been a predicted change from a 2-year to a 1-year life cycle since the 1950s.

Observed spatial and temporal patterns of outbreaks and parasite abundance largely supported model predictions. Elaphostrongylosis was first reported in the south Varanger region (far north of Norway, ~70°N, 28°E) in the early 1960s, when outbreaks killed an estimated 60–70% of calves in affected herds [(27), in 2]. This coincided with an increase in predicted climatic suitability above 1. Higher observed prevalence of *E. rangiferi* infection in reindeer grazed along the Alta-Fjord (northern Norway, approximately 70°N, 23.5°E) in the 1960s compared with the surrounding higher altitude regions is also largely replicated by model predictions, whereby the Alta-Fjord appears as an isolated hotspot (Figure 2; 1960s panel). However, observed “moderate” prevalences in the peninsulas immediately to the East and West of the Alta-Fjord was not reflected in the model. Outbreaks throughout Finnmark [northern Norwegian region lying east of the 22°E line; reviewed by (2)] in the 1970s are reflected in an increase in the range of Thermal Suitability Indices >1 in this region (Figure 2, 1970s panel). Elevated predicted thermal suitability in the Trøndelag region (central Norway, ~63.5°N) over the past decade (Figure 2, 2000s and 2010s panels, Thermal Suitability Index 1–3) is supported by recent persistent outbreaks in this region [(2), unpublished data]. These outbreaks were thought to be a result of the unusually warm summer of 2018 and were unusual in that reindeer of all ages were affected (10), suggesting significant transmission of L3 early in the spring-summer grazing season. Finally, the Norwegian Veterinary Institute diagnosed a 1-year old reindeer with elaphostrongylosis in June 2020 in Nordland, Norway (67°N). Veterinarians hypothesize that this is due to transmission early in the spring of 2020, and is, as far as the authors are aware, the earliest diagnosis (in the calendar year) of elaphostrongylosis in a 1-year-old reindeer. This is consistent with the trend toward increasing thermal suitability over time, under the assumption that this would lead to a similar trend toward earlier opportunities for transmission during the spring. Although coastal areas in this region are predicted to have been suitable for *E. rangiferi* development since the 1950s, the area of thermal suitability has expanded since the 2000s (Figures 2, 3).

The implications of our predictions for reindeer and their herders is potentially significant. Eighty percent of Norway's semi-domesticated reindeer are herded by the Sámi population in Finnmark county (3), which is the region predicted to have been most affected since 1950, and new outbreak foci have emerged in recent years (10). The predicted current thermal suitability could be used to raise awareness of the potential for *E. rangiferi* transmission on a broad spatial scale, aiding veterinary diagnoses and surveillance.

Although data available for model validation were scarce, the data that were available highlighted the fact that ambient temperature is not the only factor driving this complex system and the need for an improved understanding of the spatial epidemiology of infection. In particular, there is a paucity of data on the distribution and prevalence of *E. rangiferi* in Sweden and Finland, and it is unknown to the authors whether outbreaks similar to those observed in Norway in 2018-2019 also occurred in Sweden and Finland. Although case reports and veterinary diagnoses are often biased toward unexpected events, and therefore the lack of data cannot be taken as an indication of the absence of *E. rangiferi* in Sweden and Finland, further investigations are needed to determine the reason for the lack of support for the high predicted thermal suitability in these countries.

Competent intermediate hosts are widespread [e.g., (28)], but their distributions may not coincide with that of reindeer and other susceptible hosts. Therefore, some areas predicted to be potentially suitable for transmission from gastropod to reindeer based on ambient temperatures may not, in fact, be suitable for completion of the life cycle, for example if moisture requirements are suboptimal for the persistence of the intermediate hosts. Further studies on the ecology and habitat requirements of *E. rangiferi* intermediate hosts [e.g., (29)], and ultimately the development of informative, predictive ecological niche models for a range of the most common intermediate hosts [e.g., (30)], would allow areas unsuitable for transmission to be masked (removed) from future analyses, and could reveal important host-parasite environment interactions. Previously, a degree-day model for *Shistosoma mansoni* paired with a species distribution model for the intermediate host snail predicted important differences in the impact of climate change on the range of the intermediate host and parasite thermal suitability, moderating spatial transmission potential (31).

Similar improvements could also be made with respect to the distribution of the definitive host. Throughout the central and northern regions of Sweden and Norway, reindeer herders practice nomadic pastoralism, whereas in the south of Norway and in Finland herded and wild populations are more stationary. This diversity of grazing patterns will impact on seasonal transmission dynamics. Masking regions that are not grazed by reindeer (e.g., using reinbase.no data) and differential analysis of summer and winter degree-day estimates, taking into account L1 shedding patterns, would improve model predictions.

The use of degree-day models has been widespread in veterinary epidemiology, including for parasites with gastropod intermediate hosts [e.g., *Fasciola hepatica*–(32); *Schistosoma mansoni*–(31)]. Of particular relevance to this study is a degree-day model developed for *U. pallikuukensis* affecting muskoxen in the Arctic (21, 33). This model offered important insights into the application of a degree-day model to *E. rangiferi*. First, that soil surface temperatures may be several degrees higher than ambient temperatures, impacting on accumulated degree-days (21). As is often the case, historic soil surface temperatures were not available for the present study, nor are they typically available for future climate projections. Therefore, air temperature was used in the present study, while acknowledging that the model may underestimated accumulated degree-days as a result. Secondly, *E*. rangiferi's indirect life cycle involving a mobile, ectothermic intermediate host presents a potential problem as the intermediate host may seek out microclimate closer to its thermal optimum, impacting on accumulated degree-days. Kutz et al. (21) offered a pragmatic solution to this, placing an upper limit on the temperature at which degree days accumulate, which we also adopted in our model. Finally, Kutz et al. (33) developed a supplementary model to estimate L3 availability at a range of temperatures and two L3 and intermediate host mortality scenarios. This acknowledges a limitation of degree-day models, whereby increasing mortality rates at increasing temperatures are not considered. This may explain the high Thermal Suitability Indices we predicted in regions where elaphostrongylosis is seldom reported, and justifies the inclusion of both development and mortality rates in future models developed for *E. rangiferi*.

Despite the potential limitations of degree-day models, they offer a useful framework for climate impacts modeling, particularly for species where there are limited data to support more detailed mechanistic models. Recently, the *U. pallikuukensis* degree-day model was extended to generate an index of transmission potential similar to the thermal suitability index reported here (34). The model was also adapted for a second species, *Varestrongylus eleguneniensis* and used support empirical evidence of climate driven range expansion of these two species in the Canadian Arctic Archipelago. This demonstrates the considerable potential of degree-day models such as the one presented here to be adapted to other species of interest, such as *E. cervi, and E. alces* or *Varestrongylus alces* which are prevalent in red deer (*Cervus elaphus*) and moose (*Alces alces*) populations respectively, and may also be susceptible to climate-driven changes to their epidemiology (5, 35).

The model presented here lends support to the hypothesis that climate warming is driving changes in *E. rangiferi* epidemiology. Further work is needed to assess how predicted thermal suitability corresponds to outbreak risk, and incorporate aspects of climate and host-parasite ecology other than temperature. Addressing the knowledge gaps described above would support the development of improved risk assessment tools, which take into account eco-epidemiological processes and track real-time weather data.

## Data Availability Statement

The datasets presented in this study can be found in online repositories (36). The names of the repository/repositories and accession number(s) can be found in the article/supplementary material.

## Author Contributions

HR, TM, and RD conceived the project. HR developed the model. HR, TM, DW, and RD contributed to the interpretation of model output, writing and approval of the manuscript. All authors contributed to the article and approved the submitted version.

## Conflict of Interest

The authors declare that the research was conducted in the absence of any commercial or financial relationships that could be construed as a potential conflict of interest. The reviewer AO declared a past co-authorship with one of the authors TM to the handling editor.
